# Evaluation of oxidative stress in Insulin Dependent Diabetes Mellitus (IDDM) patients

**DOI:** 10.1186/1746-1596-2-22

**Published:** 2007-07-01

**Authors:** Vadde Ramakrishna, Rama Jailkhani

**Affiliations:** 1 Department of Biochemistry, Shri B.M. Patil Medical College, Bijapur 586 103, India; 2 Department of Biotechnology, Sri Krishnadevaraya University, Anantapur 515 003, India

## Abstract

**Background:**

Free radical mediated oxidative stress is mainly involved in the pathogenesis of diabetic complications. Proteins and lipids are among the prime targets for oxidative stress. In the present study, we evaluated the oxidative stress in chronic IDDM patients by estimating the lipid peroxidation, protein oxidation, and antioxidants status.

**Subjects and design:**

A total of 35 (15 IDDM + 20 normal healthy) children were examined in the study and estimated the lipid peroxidation, protein oxidation, and antioxidants – vitamin A (β-carotene, retinol), vitamin C, vitamin E and enzymatic antioxidants and nitric oxide.

**Results:**

A statistically significant higher values of protein carbonyl groups and MDA as lipid peroxides were observed in diabetic patients with slight reduction in the synthesis of nitric oxide. It is interesting to note that there was a decrease in the antioxidant levels with corresponding increased protein and lipid oxidation. On PAGE under native conditions, we observed decreased levels of proteins – albumin, transferrin, ceruloplasmin and heptoglobulins and variable GC globulin fractions in IDDM compared to normal healthy controls.

**Conclusion:**

Hyperglycemia induces the overproduction of oxygen free radicals and consequently increases the protein oxidation and lipid oxidation. A significance difference in the mean plasma concentration of total antioxidant status was observed in IDDM patients. The findings of the present study suggest that diabetes in an altered metabolic state of oxidation-reduction and that it is convenient to give therapeutic interventions with antioxidants.

## Background

Free radicals are very reactive chemical species, can cause oxidation injury to the living beings by attacking the macromolecules like lipids, carbohydrates, proteins and nucleic acids. Under normal physiological conditions, there is a critical balance in the generation of oxygen free radicals and antioxidant defense systems used by organisms to deactivate and protect themselves against free radical toxicity [1,2]. Impairment in the oxidant/antioxidant equilibrium creates a condition known as oxidative stress. Oxidative stress is known to be a component of molecular and cellular tissue damage mechanisms in a wide spectrum of human diseases [3-5].

Diabetes is associated with a number of metabolic alterations and principal among these is hyperglycemia. The precise mechanism by which hyperglycemia may contribute to the development of coronary heart disease (CHD) is a matter of some controversy. Known sequelae of hyperglycemia such as cellular damage, increased extra cellular matrix production and vascular dysfunction have all been implicated in the pathogenesis of vascular disease in type I and type II diabetes [3,5-10]. Mechanisms involved in the increased oxidative stress in diabetes include not only oxygen free radical generation due to nonenzymatic glycosylation (glycation), autooxidation of glycation products, but also changes in the tissue content and activity of antioxidant defense systems. Increased levels of the products of oxidative damage to lipids have been detected in serum of diabetic patients, and their presence correlates with the development of complications [6,10-15]. A variety of natural antioxidants exist to scavenge oxygen free radicals and prevent oxidative damage to biological membranes. One group of these antioxidants is enzymatic (intracellular), which include super oxide dismutase, glutathione peroxidase and catalase. In addition to enzymatic antioxidants, the major natural antioxidants, most of them derived from natural sources by dietary intake are vitamin A, vitamin C and vitamin E and carotenoids. Also, numerous small molecules are synthesized or produced within the body that has antioxidant capacity (e.g. glutathione and uric acid) [3,6,14-18].

There are several studies evaluated the free radical induced lipid peroxidation and the antioxidants in diabetic patients. Many of these studies assessed individual antioxidants that act cooperatively *in vivo *to provide greater protection to the organism against free radical damage than could be provided by any single antioxidant acting alone. Controversial reports have been reported concerning the antioxidant status in diabetic patients [17,19-21]. Protein oxidation, in contrast to lipid peroxidation, does not have the features of chain reactions. The plasma proteins destructed by peroxidation have a quite long period. Therefore, the evaluation of protein oxidation (PCG) in plasma is a respected marker of free radical intensity. There are only a few reports regarding the protein oxidation in various other pathogenic conditions and no reports are available for that processes along with antioxidants in type I diabetic patients. The aim of the present study was to evaluate the free radical reaction intensity in chronic diabetic IDDM patients on basis of protein carbonyl groups of proteins, lipid peroxidation and the status of antioxidants in plasma.

## Subjects and methods

### Subjects

A total of 35 (15 IDDM + 20 normal healthy) children were examined in the study. Based on preliminary survey, all 15 patients are treated but controlled who had the high levels of blood glucose and glycosylated hemoglobin (Gly-Hb), well above the normal ranges, were selected for the study. These children were treated with insulin, had no other medications and they had no supplemental intake of vitamins or other nutrients. The remaining 20 age-matched healthy children were chosen from the community and used as control subjects. Non-fasting heparinized venous blood samples were collected from each subject with their/parental prior consent.

The total plasma proteins were measured by biuret method, plasma albumin was measured by a colorimetric method based on the bromocresol green dye at pH 4.2 giving a colored complex [22]. Protein Oxidation was evaluated by measuring carbonyl group content in plasma proteins is a marker of free radical activity. It was measured with use of Levine method [23]. 100 μl of plasma was incubated with a 100 μl of 20 mM 2, 4-dinitrophenylhydrazine (DNPH) for 60 min. Subsequently, the protein was precipitated from the solution with the use of 20 % trichloroaceticacid. Then it was washed three times in the solution of ethanol and ethylacetate and dissolving in 1 ml of 6 M guanidine HCl in 60°C. The carbonyl group content was evaluated in a spectrophotometer at wavelength 360 nm. The results were expressed as μmol/mg of protein. The lipid peroxidation in terms of MDA was estimated by using TBARS method [24].

From all subjects, 5 – 8 ml of blood was collected in heparinized tubes at 8.00 AM after an overnight fast and immediately centrifuged at 1500 × g for 15 min at 4 C. Plasma and pelleted RBC were separated stored in eppendorf tubes and kept at -80 C until analysis. We measured SOD, GPx and catalase activity in plasma according to the methods of Beutler [25], Flohe and Gunzler [26] and Renu et al. [27]. The vitamin A and β-carotene, ascorbic acid, α-tocopherol and glutathione spectrophotometrically determined by Carr-Price reaction, DNP method and TLC methods [24]. Nitric oxide estimated in plasma by the method of Moshage et al. [28]. Electrophoresis was carried out in 10% polyacrylamide slab gels, according to the method of Laemmli [29].

### Statistical analysis

Statistical analysis was performed by Minitab software. Subjects with IDDM were compared with healthy controls. Means and standard error of means were calculated and differences between means were student's t-test. The strength of association between pairs of variables was assessed by Pearson correlation coefficient. The level of significance was set at p < 0.05.

## Results

The biochemical particulars of the study subjects including IDDM and non-diabetic controls are given in table 1. The subjects with diabetes had duration of the disease ranged from 5 – 10 years. They all were being treated with insulin. Despite of their treatment, they had hyperglycemia having blood glucose levels more than 3-fold higher than in healthy subjects. The lipid profile of IDDM is statistically insignificant changes with nondiabetics. However, the TBARS (lipid peroxidation) levels increased by 4 fold in diabetics compared to healthy controls (table 2). No significant differences were found between patients with type 1 diabetes and control subjects in the concentration of total, HDL, and LDL cholesterol and triglycerides in age and in BMI.

**Table 1:** Biochemical characteristics of normal and IDDM patients

Sl. No	Clinical data	Normal	IDDM
1.	Number	20	15
2.	Age	5 – 20	5 – 20
3.	Body mass index (BMI)	18.14 ± 2.4	18.92 ± 3.4
4.	Blood glucose	80 ± 12	342 ± 56^$^
5.	Serum total protein	6.85 ± 0.96	5.60 ± 0.6^$^
6.	Serum albumin	3.84 ± 0.45	2.80 ± 0.4^$^
7.	A:G ratio	1 – 2	0.92 ± 0.12
8.	Triglycerides	110 ± 34	153 ± 32
9.	Cholesterol	172 ± 31	187 ± 23
10.	VLDL – cholesterol	23 ± 5.6	34 ± 5^$^
11.	LDL – cholesterol	102 ± 14	121 ± 14
12.	HDL – cholesterol	38 ± 6	34 ± 8
13.	Blood urea	31 ± 8	42 ± 10
14.	Serum creatinine	1.10 ± 0.3	1.62 ± 0.42
15.	Glycosylated Hb	6.12 ± 0.3	11.5 ± 3.2

Results are expressed as mean ± SD. ^$ ^p < 0.05 against the control values

**Table 2:** Effect of IDDM on the levels of Oxidative stress parameters

Sl No.	Oxidative parameter	Control	IDDM
1.	Lipid peroxidation (TBARS)	60 ± 10	231 ± 24^$^
2.	Protein oxidation (PCG)	3.42 ± 0.5	12.2 ± 2^$^
3.	Nitric oxide	34 ± 5.4	86 ± 6.5^$^

Results are expressed as mean ± SD. ^$ ^p < 0.05 against the control values

The protein oxidation (PCG) in IDDM subjects was increased 3.5 fold with decreasing the plasma levels of total protein, albumin, globulin and their ratio compared to non-diabetic subjects. In native PAGE (not shown), the diabetic children had also decreased plasma levels of ceruloplasmin, ceruloglobulin and retinol binding protein (RBP), which are the carrier proteins for copper and vitamin A, respectively and also observed the decreased levels of proteins – albumin, transferrin, and heptoglobulins and variable GC globulin fractions in IDDM compared to normal healthy controls. Table 3 shows a significant decrease in the activities Cu-Zn super oxide dismutase, glutathione peroxidase and catalase in children with IDDM compared to controls. The glutathione peroxidase in the whole blood was also decreased in diabetic children, but their differences with those of the non-diabetic counterparts were not statistically significant. The antioxidant activity containing vitamins retinol, β-carotene, vitamin C and vitamin E were significantly decreased in plasma by 25 – 50 % respectively in diabetic children.

**Table 3:** Effect of IDDM on the blood levels of antioxidants

Sl No.	Antioxidant	Control	IDDM
1.	Vitamin A (retinol)	2.24 ± 0.25	1.35 ± 0.32^$^
2.	β-carotene	2.86 ± 0.34	1.83 ± 0.4^$^
3.	Vitamin C (Ascorbic acid)	68 ± 14	38 ± 10^$^
4.	Vitamin E (α-tocopherol)	20.4 ± 0.8	15.2 ± 1.2^$^
5.	Glutathione peroxidase (GPx)	52 ± 5	38 ± 4^$^
6.	Superoxide dismutase (SOD)	1620 ± 80	1240 ± 68^$^
7.	Catalase	256 ± 32	178 ± 28^$^
8.	Uric acid (mmol/l)	0.15 ± 0.05	0.10 ± 0.02^$^

Results are expressed as mean ± SD. ^$ ^p < 0.05 against the control values

## Discussion

Diabetes mellitus is a chronic, systemic, metabolic disease defined by hyperglycemia and characterized by alterations in the metabolism of carbohydrate, protein and lipid. Oxidative stress thought to be increased in a system where the rate of free radical production is increased and/or the antioxidant mechanisms are impaired. In recent years, the oxidative stress-induced free radicals have been implicated in the pathology of IDDM [14,19,30-34].

The present study was examined the changes in both extra and intracellular antioxidants and oxidant status in a children suffering from IDDM. The diabetic children were treated with insulin and yet they were hyperglycemic. Prolonged exposure to hyperglycemia and consequent non-enzymatic posttranslational modification of proteins resulting from chemical reaction between glucose and primary amino groups of proteins – glycation, and also increased oxygen free radicals through auto oxidation of glucose [15,33-36]. Our results are in accordance with those of previous finding clearly show the increased glucose levels induces diabetes, the overproduction of oxygen free radicals and consequently increases the protein oxidation and lipid oxidation (table 2). Plasma MDA and PCG levels were significantly higher, which would indicate that free radical mediated oxidative damage of lipids and proteins is produced at in diabetics [35,42,42]. To our knowledge, there are no reports in the literature concerning plasma PCG levels in IDDM patients in relation with antioxidant status. Carbonyl group formation is considered an early and stable marker for protein oxidation. Oxidized proteins constitute a substantial fraction of the catalytically inactive or less active forms of enzymes, which may have direct metabolic consequences [37-41]. According to Gliesner et al [35] showed no statistically significant differences were found for any of the oxidative stress markers (PCG) assessed between patients with DM1 and controls. In addition, weight, height, and routine metabolic tests, including creatininemia and cholesterol levels, were similar between the groups. The lack of significant differences between healthy controls and patients with DM1 suggested that treatment is able to counteract the increase in free radical production. Ahmed et al [41] observed profound increases in proteolytic products of glycated and oxidised proteins in diabetic patients, concurrent with much lower increases in protein glycation and oxidation adduct residues.

Nitric oxide (NO) is an important vascular target for ROS. Superoxide neutralizes NO, and the peroxynitrite formed is a source of hydroxyl radicals that can cause endothelial damage [43]. Increased levels of nitric oxide were observed in Type I DM (table 2). Astaneie et al [44] have shown the elevated levels of NO with total antioxidant power. Existence of increased total antioxidant power in the presence of normal lipid peroxidation in plasma of type I diabetic patients indicates the existence of oxidative stress. Oxidative stress therefore diminishes vessel endothelium-dependent relaxation, which is apparent in some experimental preparations even after acute exposure to hyperglycemia. Defective endothelium-dependent relaxation has been observed in chronic diabetic animals, and also in type 1 and type 2 diabetic subjects [41,45,46] and is an important potential target for antioxidant treatment.

The relationship between hyperglycemia and oxygen free radicals is supported by our results demonstrating an association between blood levels of glucose and enzymatic oxidants such as super oxide dismutase and glutathione peroxidase, only in children with IDDM (table 3). The decreased erythrocyte Cu/Zn-SOD and catalase activity in our young diabetic patients also supports the hypothesis of radical mediated injury in this disease. These results are in agreement with others [47-49]. Circulating RBC act as a sink for free radicals. Consequently, erythrocytes are subject to a continuous flux of O_2 _and H_2_O_2_. It is, therefore, possible that SOD may have an important physiological role in combating this process, since this enzyme can catalyze the dissimulation of two super oxide radicals into H_2_O_2_. In the present study, on native PAGE, the plasma concentration of ceruloplasmin in IDDM was significantly lower than those of controls. These results are in agreement with others [50] who reported a significant reduction in the plasma concentration of ceuloplasmin in type I diabetic patients. It has been reported that ceruloplasmin is immunologically altered with decrease in enzymatic oxidants. These results may explain the significant reduction in SOD activity in IDDM patients, since increased H_2_O_2 _and decreased ceruloplasmin [51]. Selenium-dependent glutathione peroxidase (GPx), which works in parallel with SOD, protects cell proteins and cell membranes against oxidative damage. In the present sudy, the GPx activity was decreased significantly compared to that of the controls and a negative correlation coefficient between GPx activity and blood glucose concentration was observed in these children (table 3). However, in the published lieterature, the GPx response to diabetes has been conflicting. Diabetics have been reported to be associated with increased [48,52] decreased [53] or unchanged [54]. In lieu with GPx, the catalase activities were also decreased in diabetes compared to control subjects (table 3). The low GPx activity could be directly explained by either low GSH content or enzyme inactivation under sever oxidative stress.

In addition to antioxidant enzyme activities, the capacity of the antioxidant system to cope with or trap the free radicals generated under normal or pathological conditions was evaluated by measuring the level of total antioxidant status. It reflects the status of α-tocopherol, vitamin A, β-carotene, ascorbic acid, albumin, uric acid and other antioxidants [42,49,57]. These extracellular nonenzymatic antioxidants delay or inhibit the oxidative process. Enhanced lipid peroxidation increases the need for lipid soluble antioxidants, such as α-tocopherol and vitamin A and β-carotene. A significance difference in the mean plasma concentration of total antioxidant status was observed in IDDM patients (table 3). Low levels of vitamin E are associated with increased incidence of diabetes and some research suggests that people with diabetes have decreased levels of antioxidants [32,42,55]. People with diabetes may also have greater antioxidant requirements because of increased production of free radicals in hyperglycemia [56]. The results of Martin-Gallan et al [42] clearly show systemic peroxidative damage associated with insufficient defense mechanisms against ROS to be already present at clinical onset of type 1 diabetes mellitus in children and adolescents. The present study has also demonstrated a significantly lower plasma ascorbate children compared with their controls. A significant negative correlation coefficient between blood glucose and vitamin C has been observed. Patients with diabetes or the metabolic syndrome have low levels of the antioxidant vitamin C [58,59] and also control the diabetes [33]. Chen et al [59] shown that high-dose oral vitamin C partially replenishes vitamin C levels in patients with Type 2 diabetes and low vitamin C levels but does not improve endothelial dysfunction or insulin resistance.

The findings of the present study suggest that diabetes in an altered metabolic state of oxidation-reduction and that it is convenient to give therapeutic interventions with antioxidants. The loss of antioxidant capacity is statistically associated with accelerated aging processes in diabetic patients, due to an increase in basal oxidation products of etrythrocytes associated with monosaccharide autooxidative glycation [4,11,18,60]. Our present work takes into account the hypothesis involving the relation between individual components in the intact clinical model and the complex oxidant-antioxidant plasmatic systemic balance. We clearly identified antioxidant markers that were affected in the presence of IDDM; include SOD, plasma albumin, ascorbic acid and α-tocopherol. Theoretically, one should be able to reverse these effects through dietary supplementation, especially of vitamin C and E.

## Abbreviations used

IDDM – Insulin dependent diabetes mellitus, PAGE – Polyacrylamide gel electrophoresis, MDA – Melondialdehyde, PCG – Protein carbonyl groups, TBARS – Thiobarbutyricacid reactives species, TLC – Thin layer chromatography, GPx – Glutathione peroxidase, NO – Nitric oxide, SOD – Superoxide dismutase.
